# Case Report: Renal embolism and acute lower extremity arterial embolism complicated by acute compartment syndrome caused by the detachment of the main body of cardiac myxoma in a child

**DOI:** 10.3389/fsurg.2025.1612421

**Published:** 2025-08-06

**Authors:** Jianbo Xue, Yi Hong, Xiaoyi Xu, Yiming Ni

**Affiliations:** ^1^ Department of Cardiothoracic Surgery, Affiliated Jinhua Hospital, Zhejiang University School of Medicine, Jinhua, Zhejiang, China; ^2^ Department of Vascular Surgery, Affiliated Jinhua Hospital, Zhejiang University School of Medicine, Jinhua, Zhejiang, China; ^3^ Department of Cardiovascular Surgery, The First Affiliated Hospital of Zhejiang University School of Medicine, Hangzhou, Zhejiang, China

**Keywords:** cardiac myxoma, lower limb arterial embolism, renal embolism, acute compartment syndrome, pediatric cardiac tumors

## Abstract

**Background:**

Primary cardiac myxoma in pediatric patients is a rare disease, with an annual incidence of approximately 0.1–0.2 cases per million children. This report presents the case of an 11-year-old male who developed multiple peripheral emboli following the detachment of a left atrial myxoma. The patient presented to the emergency department with acute abdominal pain and sudden-onset pain and sensory loss in both legs. Physical examination indicated bilateral lower limb ischemia, which was corroborated by Doppler arterial ultrasound, revealing emboli in the abdominal aorta and bilateral common iliac arteries. Abdominal CT demonstrated patchy non-enhancing low-density areas in both kidneys and the spleen, while echocardiography identified a left atrial mass. A diagnosis of acute lower limb arterial embolism, renal embolism, and splenic embolism secondary to left atrial myxoma was established. Additionally, lower limb ischemia resulted in acute compartment syndrome. The multidisciplinary team initiated systemic anticoagulation, followed by abdominal aortic embolectomy, fasciotomy decompression, and left atrial myxoma resection. Postoperative outcomes were favorable, with no residual tumor in the left atrium and complete restoration of arterial perfusion in both lower limbs. Histopathological analysis confirmed the diagnosis of myxoma.

**Conclusion:**

Although pediatric cardiac myxoma is exceedingly rare, the clinical presentation of peripheral embolism in this case raised a strong suspicion of an embolic etiology, facilitating rapid assessment and timely intervention.

## 1 Introduction

The occurrence of left atrial myxoma in children is significantly rarer, comprising approximately 10%–15% of pediatric cardiac tumors, with an estimated annual incidence of approximately 0.1–0.2 cases per million children (1). 75% of myxomas are arising in the left atrium, commonly at the mitral annulus or the margin of the fossa ovalis in the interatrial septum (2). The remaining cases are distributed among the right atrium (20%) and the ventricles or valves (5%) (3). Despite their benign histology, sporadic myxomas exhibit a recurrence rate of 1%–3%, while familial cases demonstrate recurrence rates as high as 20%.

Transthoracic echocardiography remains the diagnostic modality of choice for cardiac myxoma. Echocardiographic findings typically reveal a solitary, hyperechoic, pedunculated mass with variable size and mobility (4). Given its therapeutic implications, differentiation from intracardiac thrombus is essential for appropriate clinical management.

Given the risk of embolic complications and severe arrhythmias, prompt surgical intervention is imperative upon the diagnosis of cardiac myxoma. The tumor's anatomical location dictates the surgical approach, influencing both intraoperative strategy and postoperative outcomes (5).

Atrial fibrillation, with or without valvular heart disease, remains the most prevalent cause of cardiogenic embolism. Other common etiologies include infective endocarditis, while cardiac tumors constitute a rare but significant source of embolic events (6).

This report presents a rare case of an 11-year-old male who developed bilateral lower limb arterial embolism, renal embolism, and splenic embolism secondary to a left atrial myxoma. Sensory deficits, including loss of skin sensation and paresthesia, were noted, accompanied by distal motor dysfunction. The distal limbs appeared cold and pale, with absent bilateral femoral artery pulses. The resultant lower limb ischemia led to acute compartment syndrome (ACS) in both calves. Notably, renal embolism attributable to cardiac myxoma is extremely rare.

## 2 Case report

An 11-year-old boy with no prior medical history presented with sudden-onset abdominal pain and acute bilateral lower limb pain without any identifiable precipitating factors. Neurological symptoms included paresthesia and mild motor impairment. Approximately 3 h after symptom onset, he was admitted to the emergency department of a local hospital.

**Figure 1:**
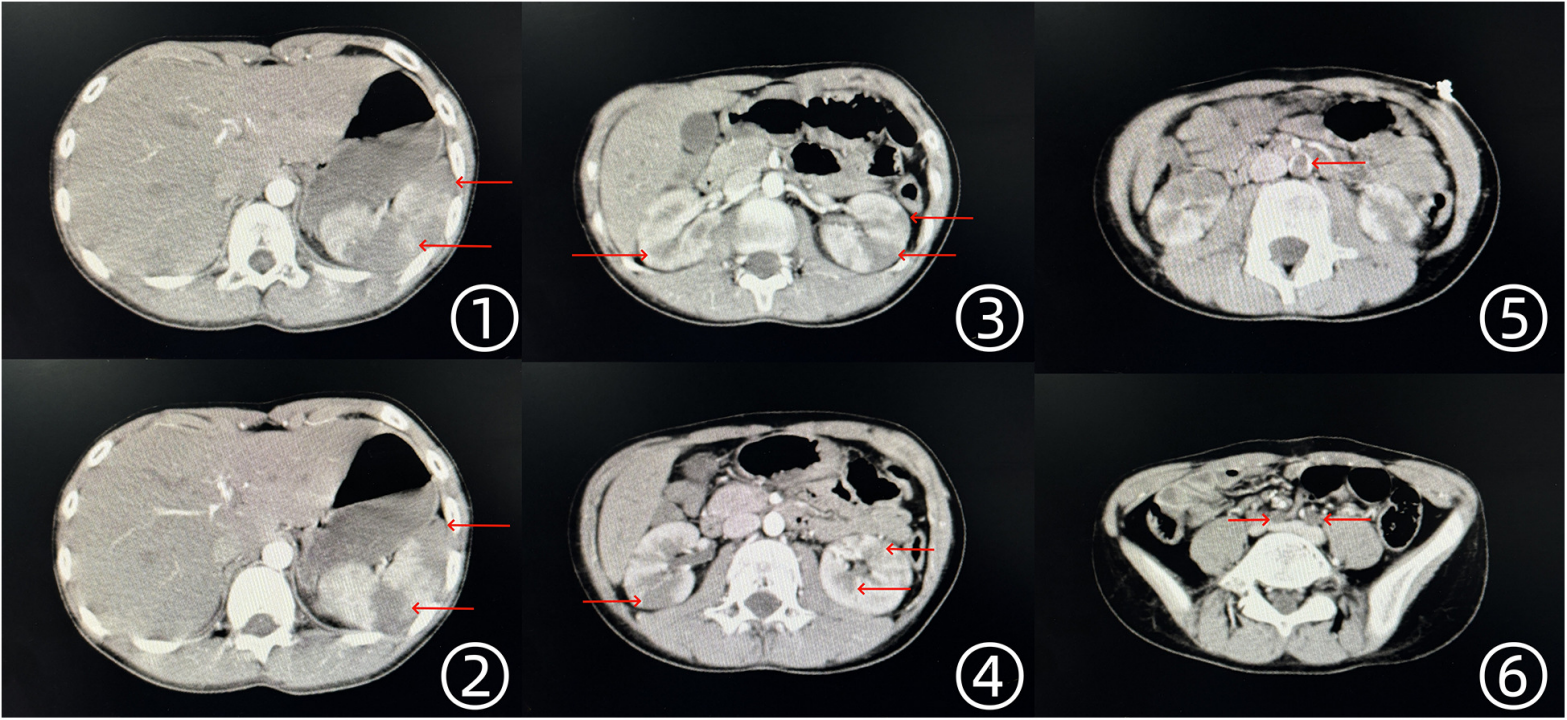
Enhanced CT scan findings of the thoracic and abdominal aorta. ①, ② Multiple low-density infarct foci in the spleen. ③, ④ Multiple low-density infarct foci in both kidneys. ⑤ Thrombus visible in the abdominal aorta. ⑥ Embolism in the bilateral iliac arteries, with absent visualization.

Laboratory investigations revealed leukocytosis (15.12 × 10^9^/L), mildly elevated high-sensitivity C-reactive protein (24.44 mg/L), and mild anemia (107 g/L), while liver and renal function tests remained within normal limits. Electrocardiography demonstrated a normal sinus rhythm without ischemic changes. Given the clinical presentation, abdominal computed tomography angiography (CTA) and color Doppler ultrasound of the lower extremities were performed. Imaging findings confirmed emboli in the abdominal aorta and bilateral iliac arteries, multiple infarctions in both kidneys, and splenic infarction (Figure 1). Doppler ultrasound further indicated an absence of arterial blood flow in both lower extremities, while venous circulation remained intact.

**Figure 2:**
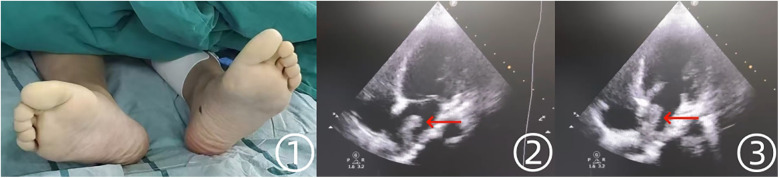
Preoperative findings of the patient's lower extremities and echocardiographic manifestations. ① The patient's bilateral lower extremities appeared pale and cold preoperatively. ②, ③ Echocardiography revealed a hyperechoic mass in the left atrium.

The patient was subsequently transferred to the emergency department of Affiliated Jinhua Hospital, Zhejiang University School of Medicine. On physical examination, the lower extremities were held in an antalgic position, with severe spontaneous pain exacerbated by passive movement. Sensory deficits, including loss of skin sensation and paresthesia, were noted, accompanied by distal motor dysfunction. The distal limbs appeared cold and pale, with absent bilateral femoral artery pulses (Figure 2 ①). No additional abnormalities were detected on systemic examination. Apart from acute pain, the patient remained in stable general condition, with no respiratory distress, hemodynamic instability, or recent history of acute illness. Skin examination revealed no lesions or abnormalities. To determine the embolic source, transthoracic echocardiography was performed, revealing a 2.9 cm × 1.3 cm hyperechoic mass with an irregular border in the left atrium, demonstrating mobility in synchrony with the cardiac cycle (Figure 2 ②,③). No significant mitral stenosis was observed, but mild mitral regurgitation was present. No additional structural abnormalities were detected.

**Figure 3:**
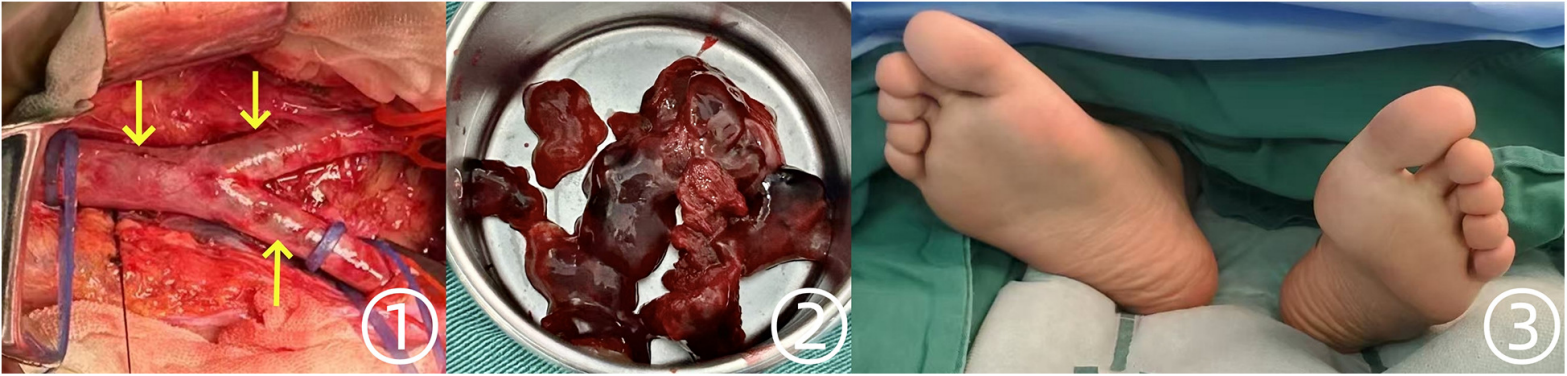
Intraoperative findings during abdominal aortic embolectomy and postoperative changes in the patient's feet. ① Saddle emboli identified in the abdominal aorta and bilateral iliac arteries. ② Gross specimens of the jelly-like tumor and thrombotic material. ③ Postoperative improvement, with feet appearing warm and ruddy.

Ultrasound confirmation of bilateral acute lower limb ischemia, along with the presence of a friable left atrial tumor, strongly indicated an embolic mechanism. Given that symptoms had persisted for over 4 h, the risk of irreversible limb ischemia and functional impairment was substantial. Consequently, systemic anticoagulation with heparin was initiated, and the vascular surgery team promptly performed an abdominal aortic embolectomy. Concurrently, embolized arterial fragments occluded by tumor material were surgically extracted (Figure 3 ①,②). The excised tumor and thrombus specimens, weighing 65 g in total, were submitted for histopathological evaluation. The tumor part measures approximately 5 × 4 × 2 cm, with a smooth surface and a translucent jelly-like appearance. It feels soft, and the overall texture is extremely fragile, prone to fragmentation. Microscopic examination revealed spindle-shaped and stellate cells embedded in myxoid and fibrinoid matrices, displaying diffuse, honeycomb-like, cord-like, and lumen-like proliferative patterns. Following successful revascularization, the patient's lower extremities exhibited restored perfusion, with the feet regaining warmth and a healthy ruddy coloration (Figure 3 ③).

During the operation, the vascular surgeon noted significant tension in both calves. Given the patient's history of arterial embolism, an intraoperative consultation with the orthopedic team confirmed a diagnosis of ACS secondary to bilateral lower limb ischemia. After obtaining informed consent from the patient's family, the orthopedic surgeon immediately performed bilateral calf fasciotomy and decompression.

Considering the exceptionally high embolic risk associated with the highly mobile and friable nature of the cardiac tumor—particularly the potential for cerebrovascular events—a multidisciplinary team determined that surgical resection was imperative. Eighteen hours after admission, the patient underwent a second fascial compartment decompression followed by atrial myxoma resection. Under cardiopulmonary bypass, the cardiac surgeon excised a translucent, gelatinous left atrial mass measuring 3 cm in diameter. The tumor exhibited high mobility and friability, with a total weight of approximately 6 g. Under microscopic observation, the tumor cells are irregular in shape with a mild cellular morphology, surrounded by halos, and distributed sparsely. The stroma is loose. Elastic fibers transversely separate the tumor pedicle from the heart wall, with the separation showing uneven density, and the stroma contains abundant blood vessels. Comparative analysis with the tumor fragments retrieved during the abdominal aortic embolectomy indicated that the embolized mass constituted the primary bulk of the tumor, while the residual left atrial lesion (3 cm × 1.5 cm) represented a smaller portion of the original neoplasm.

**Figure 4:**
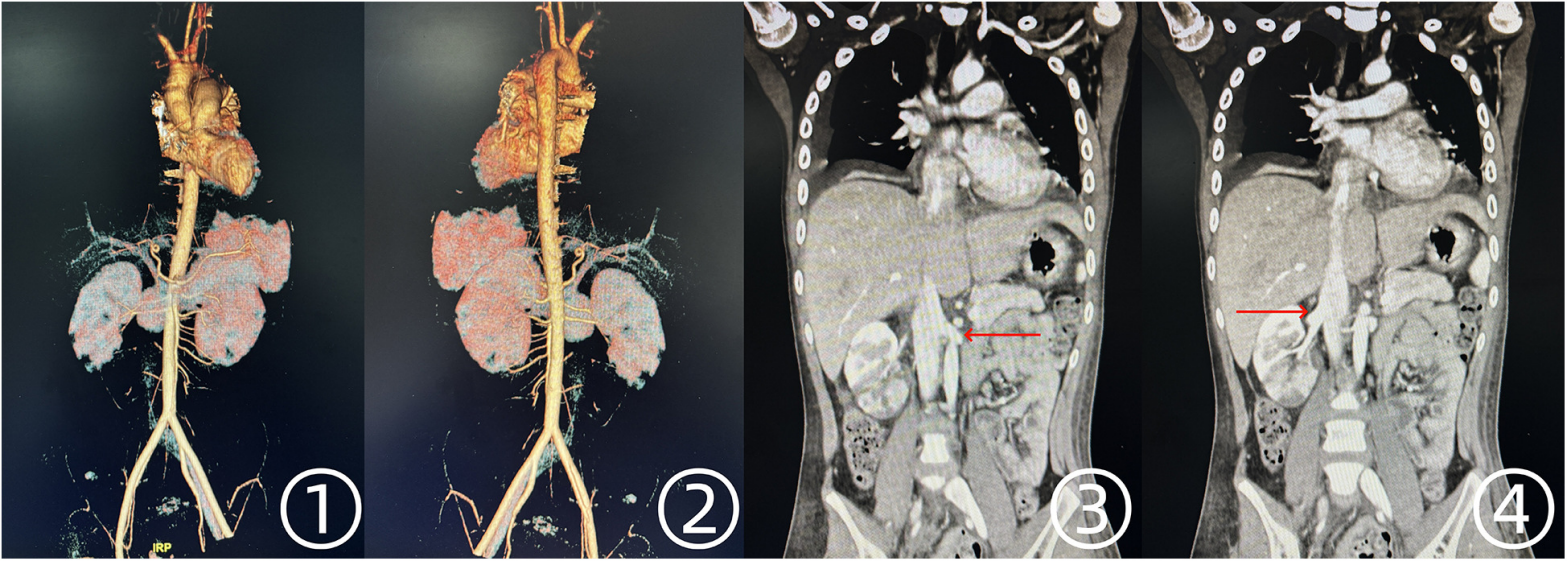
Postoperative CTA findings of the thoracic and abdominal aorta. ①, ② Unobstructed blood flow in the abdominal aorta and bilateral iliac arteries. ③, ④ Restored perfusion in both renal arteries.

Following surgery, arterial perfusion in both lower extremities was fully restored, and echocardiography confirmed the absence of residual intracardiac tumor. No signs of systemic embolism were observed postoperatively. On postoperative day 5, CTA of the chest and abdomen demonstrated no evidence of tumor embolism in other vascular territories (Figure 4).

In the early postoperative period, the patient continued to experience persistent pain and hypoesthesia in both lower extremities, with the right leg exhibiting reduced muscle strength. On hospital day 11, closed negative pressure wound drainage and debridement were performed on both lower limbs, followed by a second debridement on day 16. Gradual clinical improvement was observed, with renal function remaining stable throughout the recovery period. Palpable pulses were detected in the femoral, popliteal, posterior tibial, and dorsalis pedis arteries bilaterally. However, muscle strength in the right lower extremity remained diminished. At discharge, the right lower limb exhibited grade 4 muscle strength, while the left reached grade 5. The patient was discharged on day 18 with significant improvement in neuromotor and sensory function. Oral anticoagulation therapy was prescribed for 3 months, alongside ongoing physical rehabilitation. The potential for complete restoration of long-term motor function remains subject to further observation.

## 3 Discussion

Left atrial myxoma represents the most common primary cardiac tumor in adults, accounting for approximately 50%–75% of all cardiac tumors, with an annual incidence of 0.5–1 case per million individuals (1). In contrast, it is significantly rarer in children, comprising only 10%–15% of pediatric cardiac tumors, with an annual incidence of approximately 0.1–0.2 cases per million children (7). The overall prevalence in adults is markedly higher than in pediatric populations.

The precise etiology of cardiac myxoma remains undetermined. Genetic and immunohistochemical studies suggest that it originates from subendothelial pluripotent mesenchymal cardiac cells capable of differentiating into neural and endothelial lineages.

Cardiac myxoma is characterized by a proteoglycan-rich mucinous matrix, slow growth, and a soft, gelatinous consistency. It is attached to the cardiac chamber via a pedicle or broad base, with lengths ranging from a few millimeters to over ten centimeters, averaging 5–6 cm, and weights varying from 15 to 180 g. Morphologically diverse, it may exhibit a smooth surface or a friable texture due to surface villous projections.

Cardiac myxoma induces a wide spectrum of systemic pathophysiological effects, resulting in diverse clinical manifestations. Intracavitary growth often leads to mechanical obstruction, causing valvular stenosis or regurgitation, ultimately precipitating heart failure, which presents as chest tightness, dyspnea, palpitations, and edema (8). Tumor embolization, either via detached tumor fragments or thrombotic material, can lead to systemic embolic events, including ischemic stroke, myocardial infarction, pulmonary embolism, and arterial occlusions affecting the extremities and visceral organs (9). In addition to embolic complications, cardiac myxoma is associated with hematologic abnormalities such as hemolytic anemia, leukocytosis, and polycythemia. The tumor's presence can elicit an inflammatory response, promoting elevated levels of interleukin-6 (IL-6), C-reactive protein, immunoglobulins, and erythrocyte sedimentation rate (10), contributing to systemic symptoms such as fever, malaise, anorexia, and weight loss (11). Additionally, tumor-secreted cytokines may trigger a systemic inflammatory response syndrome in affected individuals (12). While the majority of patients exhibit symptoms prompting further cardiac evaluation, there are documented cases of asymptomatic individuals in whom cardiac masses were incidentally detected during unrelated examinations (12).

This report presents a case complicated by arterial embolism. Fortunately, prompt surgical intervention preserved function in the ischemic lower extremities, kidneys, and spleen. In cardiac myxoma, tumor size, friability, and prolapse through cardiac valves are primary risk factors for recurrent embolism, serving as critical indications for urgent surgical resection. Despite the tumor's considerable size in this case, it did not significantly impair left ventricular preload or cause notable outflow tract obstruction, which explains the absence of preceding symptoms prior to the embolic event.

Acute renal infarction (ARI) is a medical emergency characterized by obstruction of the main renal artery or its branches due to embolism or thrombosis, leading to the cessation of blood flow, ischemic necrosis, and potential irreversible renal damage (13).

Based on etiology, renal infarction is categorized into four types: cardiogenic (embolic), renovascular (secondary to renal artery injury), hypercoagulability-associated, and idiopathic (14). The reported prevalence of cardiogenic renal infarction varies across studies, with estimates ranging from 10% to 50% (14, 15). Renal infarction secondary to left atrial myxoma is a rare clinical entity.

Renal artery embolism is rarely an isolated event and is frequently secondary to underlying cardiogenic pathology. Patients with renal embolism are at heightened risk for recurrent systemic thromboembolic events, with an increased likelihood of fatal cardiovascular and cerebrovascular complications (15). Long-term anticoagulation therapy is essential to mitigate the risk of recurrent embolism and systemic thrombotic events.

In the present case, the rapid decision to perform an emergency embolectomy was critical in preventing irreversible renal damage. No time was lost to unnecessary investigations, and revascularization was achieved within 5 h of symptom onset. Delayed intervention could have resulted in significant renal impairment, underscoring the importance of timely recognition and immediate surgical management in cases of acute renal infarction.

ACS is characterized by increased intracompartmental pressure within a closed osteofascial space, leading to a reduction in tissue perfusion and subsequent ischemia and hypoxia of muscles and nerves, resulting in a series of symptoms (16). If ischemia persists, irreversible damage to muscles, nerves, and vascular endothelium ensues, potentially resulting in permanent limb dysfunction and the development of Volkmann ischemic contracture (17).

Treatment strategies for PACS: PACS constitutes a surgical emergency requiring immediate intervention to optimize clinical outcomes. Prompt and effective management significantly improves prognosis. In children with a high index of suspicion or those who have undergone conservative treatment for 2–4 h without improvement, emergency fasciotomy is warranted (18). Surgical decompression involves complete incision of all affected fascial compartments to relieve intracompartmental pressure, thereby restoring tissue perfusion and mitigating ischemic damage to muscles and nerves. In cases of extensive necrosis or crush syndrome, aggressive debridement and antimicrobial therapy are essential.

Early intervention is paramount. The conventional approach advocates performing fasciotomy within 6 h of symptom onset (19). While the early intervention principles for pediatric ACS closely align with those for adults—emphasizing timely decompression and prophylactic fasciotomy when necessary—the diagnostic and treatment window for children is comparatively longer. The timing of surgical decompression can be extended, and in cases where a delayed diagnosis has resulted in muscle and nerve damage, fasciotomy remains advisable, even in the late stages of the disease. Notably, no definitive evidence establishes a direct linear correlation between diagnostic delay and prognosis. Moreover, children exhibit a remarkable capacity for neuromuscular regeneration, with permanent nerve damage occurring infrequently. Given these factors, the benefits of surgical intervention outweigh the potential risks, supporting its recommendation in cases of delayed presentation (20).

In the present case, early recognition of concurrent osteofascial compartment syndrome and immediate surgical intervention led to a favorable outcome. Timely fasciotomy prevented irreversible neuromuscular damage, averting permanent disability and the development of Volkmann ischemic contracture.

## 4 Conclusion

This report presents a rare case of an 11-year-old male with a giant left atrial myxoma, in which tumor detachment resulted in renal artery embolism, splenic artery embolism, and bilateral iliac artery embolism. The ensuing lower limb ischemia led to the development of acute osteofascial compartment syndrome in both calves. A tumor of this size, morphology, and location is rarely documented in the literature, particularly in the pediatric population. Despite its substantial size and the presence of systemic embolic complications, timely diagnosis and emergency surgical intervention facilitated a successful recovery. Postoperatively, no recurrence of the cardiac tumor has been observed, and the patient remains asymptomatic with no residual deficits.

## Data Availability

The original contributions presented in the study are included in the article/Supplementary Material, further inquiries can be directed to the corresponding author.
